# Modified Peritoneal Fenestration as a Preventive Method for Lymphocele after Kidney Transplantation: A Preliminary Report

**DOI:** 10.3390/jcm13195878

**Published:** 2024-10-02

**Authors:** Mohammadsadegh Sabagh, Sanaz Weber, Nastaran Sabetkish, Ali Ramouz, Sanam Fakour, Christian Morath, Markus Mieth, Martin Zeier, Elias Khajeh, Arianeb Mehrabi, Mohammad Golriz

**Affiliations:** 1 Department of General, Visceral, and Transplantation Surgery, University of Heidelberg, 69120 Heidelberg, Germany; mssabagh@gmail.com (M.S.); nastaran.sabetkish@med.uni-heidelberg.de (N.S.); ali.ramouz@med.uni-heidelberg.de (A.R.); sanam.fakour@med.uni-heidelberg.de (S.F.); markus.mieth@med.uni-heidelberg.de (M.M.); elias.khajeh@med.uni-heidelberg.de (E.K.); arianeb.mehrabi@med.uni-heidelberg.de (A.M.); 2 Department of Anesthesiology, Heidelberg University Hospital, 69120 Heidelberg, Germany; sanaz.weber@gmail.com; 3 Department of Nephrology, Heidelberg University Hospital, 69120 Heidelberg, Germany; christian.morath@med.uni-heidelberg.de (C.M.); martin.zeier@med.uni-heidelberg.de (M.Z.); 4 Department of General and Visceral Surgery, Diakonie Clinic Jung-Stilling, Wichernstraße 40, 57074 Siegen, Germany

**Keywords:** kidney transplantation, lymphocele, prevention and control

## Abstract

**Background**: We aimed to assess the safety of a modified peritoneal fenestration technique with clipping of the window edges during kidney transplantation (KTx) and to determine its impact on reducing lymphocele following KTx. We compared the outcomes of this modified method with those of peritoneal fenestration without clipping. **Methods**: Among 430 consecutive KTxs performed between 2015 and 2019, preventive peritoneal fenestration and clipping of the margins were performed in 25 patients. These patients were compared with 75 matched patients in whom the margins were not clipped. Postoperative lymphocele formation and other patient data were compared between these two groups. **Results**: The rate of clinically relevant lymphocele decreased by 2.7% after peritoneal fenestration with clipping, although this decrease was not statistically significant (*p* = 0.829). There was no significant increase in the rate of other complications in the modified fenestration group (*p* = 0.067). The incidence of clinically significant lymphocele formation was notably higher in patients with a body mass index greater than 25 kg/m^2^ (*p* = 0.028). Univariate analysis indicated that older recipients, individuals with a history of previous abdominal surgery, those receiving the kidney from deceased and older donors were at increased risk of developing a clinically relevant lymphocele. **Conclusions**: Our preliminary results suggest that peritoneal fenestration with clipping may be as effective as the conventional fenestration technique in preventing lymphocele formation. Further clinical trials with larger sample sizes are required to determine the exact role of preventive peritoneal fenestration with clipping in preventing clinically relevant lymphocele after KTx.

## 1. Introduction

Lymphatic complications after kidney transplantation (KTx) are still a challenging surgical problem, with an incidence rate between 0.6 and 51% [1,2,3]. Lymphocele is any collection of fluid near the transplanted kidney that is not a urinoma, hematoma, or abscess [4,5]. Although asymptomatic lymphoceles need no treatment, symptomatic lymphoceles can result in serious morbidities because they compress adjacent structures. Therefore, lymphocele prophylaxis has been an important discussion point among transplant surgeons since KTx was introduced.

Several methods have been reported to prevent lymphocele development after KTx, including ligation of lymphatic vessels in the donor and recipient [6], insertion of a drain during surgery [7], use of polymeric sealants or hemostatic biomaterials [8,9], and peritoneal fenestration. Several studies have described the advantages and disadvantages of these preventive methods [10]; however, the reported outcomes are diverse, and the best approach has still not been determined.

In peritoneal fenestration, the lymphocele is drained into the peritoneal cavity by opening the peritoneum (fenestration) [5,11]. This prophylactic fenestration has been reported to prevent lymphocele formation after KTx [5,12]. However, while an earlier study in our center showed no significant reduction in the rate of lymphocele after KTx with prophylactic fenestration, although we did observe lower rates of clinically relevant lymphocele, which need interventional or surgical therapy (grade B and C) following peritoneal fenestration [13]. Our previous study demonstrated a significant reduction in the incidence of Type C lymphoceles using preventive fenestration (5.3% vs. 8.8%) [13]. The high rate of lymphocele following fenestration might be explained by the size of the fenestration; a small fenestration may cause the opening to close early on, and a lymphocele to develop. However, increasing the size of the fenestration can lead to intestinal hernia and can complicate kidney biopsy procedures [10].

To avoid the complications associated with larger fenestrations, we have modified peritoneal fenestration to include edge clipping of the opening to stabilize it without enlarging the size. This technique has been used to prevent lymphocele recurrence after laparoscopic lymphocele fenestration [14] but has not been used as a prophylactic method. In this study, we evaluated the safety of our modified peritoneal fenestration technique during KTx and assessed whether it reduces the rate of lymphocele formation and other complications compared with the unmodified technique without clipping.

## 2. Materials and Methods

### 2.1. Patients and Methods

Since 2008, peritoneal fenestration has been the method of choice for preventing lymphocele after KTx at Heidelberg University Hospital. Since 2015, our modified peritoneal fenestration technique with edge clipping was implemented in our center. Between January 2015 and June 2019, this method was used on 25 patients out of a total 430 adult kidney transplantations from living and deceased donors (excluding multi-organ transplantations or intraperitoneal KTxs). Data of all patients were retrospectively screened from a prospectively maintained database. To assess the impact of peritoneal fenestration and clipping, data from all patients (n = 25) who underwent peritoneal fenestration and clipping between January 2015 and June 2019 were analyzed. No formal sample size calculation was performed. Additionally, 75 patients who underwent conventional fenestration without peritoneal clipping were selected from our total database in a 1:3 ratio using propensity score matching. Patient data and outcomes were compared between these two groups, particularly postoperative lymphocele formation.

The study protocol received approval from the regional ethics committee of the medical faculty, with the approval number S-811/2018. The study was carried out in compliance with good clinical practice standards, and followed the ethical guidelines set forth in the Declaration of Helsinki.

### 2.2. Preoperative Data

Preoperative data included the recipient age, gender, previous abdominal surgery, American Society of Anesthesiologists (ASA) physical status, and whether the donor organ was from a brain dead or living donor.

### 2.3. Surgical Procedure

**Figure 1:**
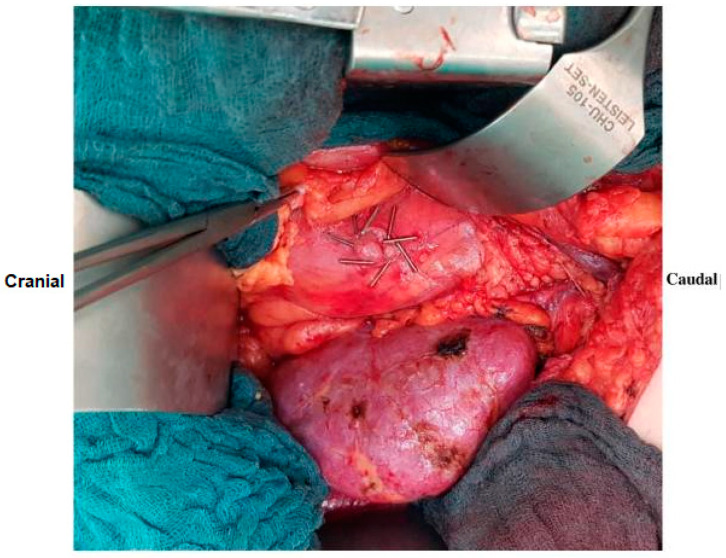
Surgical technique demonstrating fenestration with clipping [11].

The surgical procedure followed the protocol outlined in our previous publication (Figure 1) [11]. To summarize, the procedure involved an extraperitoneal surgical approach with a J-shaped (hockey-stick) incision in the abdominal wall to place the kidney graft into the left or right iliac region. After mobilizing the peritoneum from the psoas muscle and dissecting the iliac arteries and veins, the lymphatic vessels were ligated. Subsequently, the kidney vessels were anastomosed end-to-side with the common, internal, or external iliac artery/vein following the preparation of the donor organs during a back-table procedure. The ureter was anastomosed to the urinary bladder using the extravesical ureteroneocystostomy approach [15]. The medial peritoneum was fenestrated with a 2-cm window near the graft hilum. In the control group, the peritoneal edges were left unclipped, whereas in the study group, the edges of the peritoneal window (360°) were secured with eight large metal clips (Horizon, Weck Closure Systems, Research Triangle Park, NC, USA), placed every 45° in an oblique orientation [11]. All patients were equipped with a single-J stent, a suprapubic catheter, a transurethral catheter, and two easy-flow drains.

### 2.4. Intraoperative and Postoperative Data

Intraoperative and postoperative data were retrieved from the database. Intraoperative data included the graft implantation side (right or left), operation duration (in minutes), and blood loss (in milliliters). Postoperative data covered the length of stay in the intermediate care unit and hospital, complications related to lymphocele, and mortality. Lymphoceles were diagnosed and graded based on the system established by Mehrabi et al. [4]. Grade A was defined as small, asymptomatic lymphoceles that require no specific treatment or only diagnostic or therapeutic aspiration (puncture). Grade B included symptomatic lymphoceles with mild to moderate symptoms treated with non-surgical interventions, such as percutaneous external drainage, sclerotherapy, double-J stent placement, or radiation therapy. Grade C consisted of large or complicated lymphoceles with significant symptoms or complications requiring either laparoscopic or open surgical treatment. During their hospital stay and at each outpatient follow-up, all patients received daily ultrasound scans. Easy-flow drains were removed when fluid secretion diminished, provided there was no bleeding or lymphorrhea.

### 2.5. Propensity-Matching of Case and Control Groups

Propensity score matching was performed using a 1:3 nearest neighbor matching algorithm without replacement, with distances determined by logistic regression. Propensity score matching was based on recipient age, gender, ASA score, body mass index (BMI), previous abdominal surgery, and donor age. The absolute standardized mean difference in distances between patients undergoing fenestration with and without clipping was balanced after conditioning on the propensity score, where a difference of less than 0.1 in absolute standard mean difference after matching was considered an indication of good balance. Propensity match analyses were performed using R software version 4.0.0 (R Foundation) with the ‘MatchIt’ and ‘optmatch’ packages. The adequacy of matching is shown in Supplementary S1 and S2.

### 2.6. Statistical Analysis

**Figure 2:**
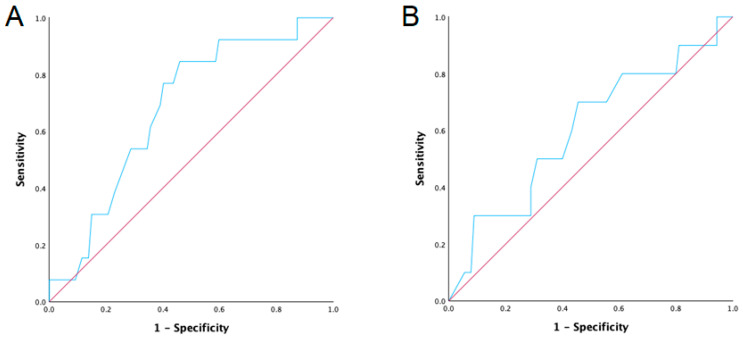
ROC Curve for calculation of cut-off value for recipients’ age (**A**). ROC Curve for calculation of cut-off value for donors’ age (**B**).

Data were analyzed using IBM SPSS Statistics for Windows, Version 22.0 (IBM Corp., released 2013, Armonk, NY, USA). Categorical data were expressed as proportions and percentages, and compared using the Chi-square test or Fisher’s exact test. Continuous data were reported as medians and ranges and compared using the Student’s *t*-test or Mann–Whitney U test. Univariate logistic regression analyses were conducted to identify independent predictors of lymphocele formation. Receiver operating characteristic (ROC) curve analysis was used to determine the optimal cut-off values for recipient and donor ages (Figure 2). Both types of analyses provided odds ratios (OR) along with 95% confidence intervals (CI). Statistical significance was defined as a two-sided *p* value of less than 0.05.

## 3. Results

After matching, a total of 100 KTxs with intraoperative peritoneal fenestration were included in this prospective analysis (25 with clipping and 75 without clipping). The median follow-up duration for the patients was 5.22 ± 2.12 years (5.90 ± 2.26 years for the peritoneal fenestration with clipping group and 5.00 ± 2.04 years for the peritoneal fenestration without clipping group; *p* = 0.066).

There were no statistically significant differences in preoperative patient characteristics, including age, gender, BMI, previous abdominal surgery, ASA, donor age, and donor type (Table 1). There was no significant difference in initial renal diseases between the peritoneal fenestration with clipping and peritoneal fenestration without clipping groups, including diabetes (8.0% vs. 2.7%, *p* = 0.239), hypertension (88.0% vs. 92.0%, *p* = 0.545), glomerulonephritis (48.0% vs. 50.7%, *p* = 0.817), and polycystic disease (20.0% vs. 18.7%, *p* = 0.883). The respective rates of hemodialysis (32.0% vs. 46.7%), peritoneal dialysis (8.0% vs. 5.3%), and no dialysis (60.0% vs. 48.0%) were not significantly different between the peritoneal fenestration with clipping and peritoneal fenestration without clipping groups (*p* = 0.430). The duration of dialysis did not differ significantly between the peritoneal fenestration with clipping and peritoneal fenestration without clipping groups (5.54 ± 5.16 vs. 5.04 ± 5.12 years, *p* = 0.788).

Analysis of intraoperative patient characteristics showed no significant differences in implantation side (*p* = 1.000) and operation time (*p* = 0.708) between the groups. However, intraoperative blood loss was slightly lower in patients who underwent fenestration with clipping (*p* = 0.048). Regarding intraoperative outcomes, there were no significant differences between the two groups in operation time (205 min in both groups, *p* = 0.708), lymphocele rate (12.0% vs. 13.3%, *p* = 0.863), clinically relevant lymphoceles (8% vs. 10.7%, *p* = 0.829), and other complications (*p* = 0.067). Postoperative overall morbidity was evaluated using the Clavien-Dindo classification. None of the patients in the fenestration and clipping group experienced complications of Clavien-Dindo grade ≥ 3b, while 10.6% of patients in the peritoneal fenestration without clipping group had complications of Clavien-Dindo grade ≥ 3b (*p* = 0.089) and were successfully managed. None of the patients in either group experienced postoperative hernia.

We compared the perioperative data between the patients with and without grade B and C lymphocele (Table 2). The proportion of patients with a BMI higher than 25 kg/m^2^ was significantly higher in patients who developed clinically relevant lymphocele (*p* = 0.028), but other perioperative factors including recipient and donor age, gender, ASA, donor type, side of graft and implantation side, previous abdominal surgery, intraoperative blood loss, and surgical time did not differ between the patients with and without clinically relevant lymphocele.

Serum blood urea nitrogen (BUN) on the 7th postoperative day was: 85.2 ± 60.5 mg/dL for the peritoneal fenestration with clipping group, and 77.8 ± 48.6 mg/dL for the peritoneal fenestration without clipping group (*p* = 0.534). Serum BUN on the 31st postoperative day was: 51.8 ± 20.7 mg/dL for the peritoneal fenestration with clipping group, and 49.8 ± 15.9 mg/dL for the peritoneal fenestration without clipping group (*p* = 0.626). Serum creatinine level on the 7th postoperative day was: 4.6 ± 6.7 mg/dL for the peritoneal fenestration with clipping group, and 3.1 ± 3.2 mg/dL for the peritoneal fenestration without clipping group (*p* = 0.131). Serum creatinine level on the 31st postoperative day was: 1.5 ± 0.5 mg/dL for the peritoneal fenestration with clipping group, and 1.4 ± 0.4 mg/dL for the peritoneal fenestration without clipping group (*p* = 0.204). Glomerular filtration rate (GFR) on the 7th postoperative day was: 39.6 ± 26.8 mL/min/1.73 m^2^ for the peritoneal fenestration with clipping group, and 43.5 ± 24.9 mL/min/1.73 m^2^ for the peritoneal fenestration without clipping group (*p* = 0.509). GFR on the 31st postoperative day was: 61.0 ± 18.7 mL/min/1.73 m^2^ for the peritoneal fenestration with clipping group, and 65.7 ± 19.0 mL/min/1.73 m^2^ for the peritoneal fenestration without clipping group (*p* = 0.289).

To evaluate the factors associated with developing a clinically relevant lymphocele, we also performed a univariable logistic regression analysis in the matched cohort (Table 3), and found that recipients who were older (OR = 7.56, 95% CI: 2.37–24.07, *p* < 0.001), who had previous abdominal surgery (OR = 1.03, 95% CI: 0.34–3.08, *p* = 0.010), and those receiving the kidney from deceased (OR = 0.18, 95% CI: 0.04–0.67, *p* = 0.007) and older donors (OR = 3.23, 95% CI: 1.08–9.64, *p* = 0.056) were more likely to develop a clinically relevant lymphocele.

## 4. Discussion

In this study, we describe the effects of a modified peritoneal fenestration technique on lymphocele formation and other outcomes following KTx. The results revealed that although clipping of the edges could decrease the formation of clinically relevant lymphocele from 10.7% to 8.5% compared with fenestration without clipping, this reduction was not statistically significant. Preventing lymphoceles after KTx is highly desirable because of their high frequency and potential adverse consequences. Peritoneal fenestration during KTx and internal drainage of lymph fluid have been used to control lymphoceles in many transplantation centers [13,16,17,18], and a systematic review showed that peritoneal fenestration reduces the rate of symptomatic lymphocele without increasing postoperative surgical complications [5]. However, this meta-analysis had several limitations, such as inconsistencies in endpoint definitions, significant selection bias, absence of standardized interventions, and inadequate follow-up periods.

Larger fenestrations can cause intestinal herniation and complicate kidney biopsy procedures. Therefore, smaller fenestrations are preferred. However, small fenestrations tend to close quickly, which increases the likelihood of developing subsequent lymphoceles [14]. Clipping the window edges during laparoscopic therapeutic fenestration has been suggested to avoid window closure in small fenestrations, and to prevent lymphocele recurrence [14]. We previously introduced the protocol of a randomized controlled trial to investigate the effect of this method on preventing lymphocele formation following KTx as a prophylactic procedure [11]. In the present cohort study, we made a 2 cm peritoneal fenestration in our patients, which did not cause postoperative intestinal herniation either with or without clipping. We showed that clipping reduced the formation of clinically relevant lymphocele by 2.7% after KTx, but that this reduction was not significant.

Different presurgical factors may affect postsurgical complications [19]. Although donor type and previous abdominal surgery have been shown to affect lymphocele formation in previous studies [20], we found no effect in this study. In addition, it remains unclear from our results if clipping the window edges during peritoneal fenestration prevents lymphocele formation.

According to our findings, age and previous abdominal surgery are associated with lymphocele formation. Age is a critical factor that can influence the likelihood of developing lymphoceles. As patients age, the tissue healing process may become less efficient, and there may be an increased risk of complications. In older patients, the decreased elasticity of lymphatic vessels and the potential presence of age-related comorbidities can contribute to the formation of lymphoceles. Additionally, the anatomical changes that occur with aging can impact lymphatic drainage and increase susceptibility to lymphocele formation [21]. Previous abdominal surgery can significantly impact the risk of developing lymphoceles. Surgical interventions can disrupt normal lymphatic drainage and create potential spaces where lymph fluid can accumulate. Scar tissue and altered anatomy from prior surgeries can impede the proper flow of lymphatic fluid, leading to lymphocele formation. Furthermore, surgeries involving the peritoneum or lymphatic system can increase the likelihood of lymph leakage and subsequent lymphocele development [22]. We also found that recipient BMI differs in Grade A versus Grade B and C lymphoceles. Typically, Grade A lymphoceles are smaller and may not cause significant symptoms, while a higher BMI can be associated with larger or more symptomatic lymphoceles (Grades B and C) [22].

Previous studies have suggested that immunosuppressive drugs are risk factors for lymphocele formation after kidney transplantation [2]. However, since we use a standard immunosuppressive therapy for all our patients, this comparison was not included in our analysis.

A limitation of this study is the risk of selection bias because of the non-randomized setting, although this was mitigated through matched-pair analysis. Selection of patients for the two groups was not influenced by patient-related factors. However, caution is needed when selecting patients for fenestration, particularly those with contraindications to hemodialysis, as fenestration prevents peritoneal dialysis because of the peritoneal breach. Additionally, while our propensity score matching aimed to balance key covariates, the mismatch in donor type between the groups could introduce a potential source of bias. To mitigate this, we recommend ensuring more balanced donor characteristics or using additional statistical techniques to account for donor type in future research. Another limitation of this study is that limited familiarity with the technique among surgeons in our clinic may reflect surgeon bias rather than patient selection bias.

To conclude, we investigated the impact of clipping versus non-clipping of fenestration on lymphocele formation rates. Our analysis revealed that the rates of lymphocele formation were not statistically significantly different between the two groups. This finding suggests that the choice to clip or not clip the fenestration does not have a substantial effect on the incidence of lymphoceles. However, this may be attributed to the small sample size in this study. To determine the true impact of clipping on clinically relevant lymphocele rates, further randomized controlled trials with larger sample sizes are necessary. We believe that clipping the window edges during peritoneal fenestration can prevent spontaneous closure in small fenestrations. Using this method, we do not need large fenestration, as it can increase the risk of internal herniation.

## Figures and Tables

**Table 1:** Preoperative, intraoperative, and post-transplantation characteristics of patients with and without clip after kidney transplantation with prophylactic peritoneal fenestration.

		All Peritoneal Fenestrations before Matching (n = 405)	Peritoneal Fenestration + Clipping (n = 25)	*p*	Peritoneal Fenestration without Clipping after Matching (n = 75)	*p*
**Age (years), median (range)**		56 (6–87)	41 (21–69)	**0.042**	45 (18–71)	0.723
**Gender, (female), n (%)**		149 (26.8)	7 (28.0)	0.375	29 (38.7)	0.471
**BMI (kg/m^2^), median (range)**		25 (16.2–41.7)	23.9 (17.4–36.3)	0.094	24.6 (17.2–35.9)	0.650
**Previous abdominal surgery, n (%)**		143 (35.3)	9 (36.0)	0.944	19 (32.0)	0.303
**ASA, n (%)**				0.441		0.285
	II	42 (10.4)	1 (4.0)		9 (12.0)	
	III	355 (87.6)	24 (96.0)		63 (84.0)	
	IV	8 (2.0)	0 (0.0)		3 (4.0)	
**Donor age (years), median (range)**		56 (6–87)	55 (37–81)	0.325	57 (12–81)	0.682
**Donor type (deceased), n (%)**		301 (74.2)	9 (36.0)	**<0.001**	43 (57.3)	0.105
**Implantation side (right), n (%)**		185 (45.7)	15 (60.0)	0.163	44 (58.7)	1.000
**Blood loss (mL), median (range)**		300 (500–1700)	300 (100–1100)	0.061	300 (100–2000)	**0.048**
**Operation time (min), median (range)**		208 (51–800)	205 (145–402)	0.828	205 (145–402)	0.708
**Total lymphocele, n (%)**		55 (13.6)	3 (12.0)	0.857	10 (13.3)	0.863
**Clinically relevant lymphocele, n (%)**		43 (10.6)	2 (8.0)	0.678	8 (10.7)	0.829

BMI: Body mass index; ASA: American Society of Anesthesiologists. Bold: *p*-values with statistically significant differences

**Table 2:** Preoperative and intraoperative characteristics of patients with and without clinically relevant lymphocele after kidney transplantation.

	Clinically Relevant (Grade B and C) Lymphocele (n = 10)	Grade A Lymphocele (n = 3)	*p*
**Recipient age (years), median (range)**	56.5 (45–71)	39 (27–56)	0.077
**Donor age (years), median (range)**	61 (34–81)	63 (53–77)	1.000
**BMI (kg/m^2^), median (range)**	24.9 (21.6–35.9)	21 (17.7–22.6)	0.298
**BMI (≥25 kg/m^2^), n (%)**	5 (50.0)	0 (0.0)	**0.028**
**Gender (female),** **n (%)**	4 (40.0)	1 (33.3)	1.000
**ASA, (>III), n (%)**	7 (70.0)	3 (100.0)	0.528
**Donor type (deceased), n (%)**	9 (90.0)	2 (66.7)	0.423
**Side of graft (right), n (%)**	6 (60.0)	1 (33.3)	0.559
**Side of kidney implantation (right), n (%)**	4 (40.0)	1 (33.3)	1.000
**Previous abdominal surgery,** **n (%)**	5 (50.0)	3 (100.0)	0.231
**Blood loss (mL), median (range)**	325 (230–1000)	500 (150–500)	0.559
**Operation time (min), median (range)**	220 (163–290)	210 (205–345)	0.917

BMI: body mass index; ASA: American Society of Anesthesiologists. Bold: *p*-values with statistically significant differences

**Table 3:** Univariate analysis of potential risk factors for the formation of clinically relevant lymphocele.

		Univariate Analysis	
	OR	95% CI	*p*
Recipient age (>55 years)	7.560	2.374–24.073	**<0.001**
Recipient gender (female)	1.038	0.349–3.089	1.000
BMI (≥25 kg/m^2^)	1.432	0.387–5.302	0.738
Previous abdominal surgery	4.507	1.497–13.568	**0.010**
Donor type (deceased)	0.181	0.048–0.677	**0.007**
Donor age (>60 years)	3.239	1.088–9.642	**0.056**
Operation time	1.008	0.997–1.018	0.160
Blood loss (>1000 mL)	1.521	0.371–6.243	0.693
Fenestration with clipping	0.837	0.166–4.221	0.826

Bold: *p*-values with statistically significant differences

## Data Availability

The raw data supporting the conclusions of this article will be made available by the authors upon request and after approval from the ethics committee.

## Supplementary Materials

The following supporting information can be downloaded at: https://www.mdpi.com/article/10.3390/jcm13195878/s1, Supplementary S1. Density plot of propensity scores. Comparing the distribution of propensity scores in both groups before and after matching, which reveals a substantial overlap post-matching. Supplementary S2. Standardized mean differences (SMD) before and after matching between groups with and without clip fenestrations. The standardized mean difference compares the means of the covariates between treated and control groups. As shown, after matching, the SMD for each covariate lies below the threshold (0.25).
