# Supplementary material for: Modified Peritoneal Fenestration as a Preventive Method for Lymphocele after Kidney Transplantation: A Preliminary Report
Source: J Clin Med. 2024 Oct 2;13(19):5878. doi: 10.3390/jcm13195878 (PMC11477912; doi:10.3390/jcm13195878)
Supplement: Supplementary file 1 [file jcm-13-05878-s001.zip › jcm-3168915-supplementary.pdf]

Modified Peritoneal Fenestration as a Preventive Method for Lymphocele After Kidney Transplantation: A Preliminary Report

Mohammadsadegh Sabagh MD[1], Sanaz Weber MD[2], Nastaran Sabetkish MD[1], Ali Ramouz MD[1], Sanam Fakour MD[1], Christian Morath MD[3], Markus Mieth MD[1], Martin Zeier MD[3], Elias Khajeh MD, MPH[1], Arianeb Mehrabi MD[1], Mohammad Golriz MD[1,4]

[1] Department of General, Visceral, and Transplantation Surgery, University of Heidelberg, Heidelberg, Germany

[2] Department of Anesthesiology, Heidelberg University Hospital, Heidelberg, Germany

[3] Department of Nephrology, Heidelberg University Hospital, Heidelberg, Germany

[4] Department of General and Visceral Surgery, Diakonie Clinic Jung-Stilling, Siegen, Germany

Corresponding author's contact information:

Professor Dr. med. Mohammad Golriz, MD, FICS, FACS

Department of General and Visceral Surgery,

Diakonie Clinic Jung-Stilling, Siegen, Germany

Wichernstraße 40, 57074 Siegen, Germany

Tel: 0271 333 47 54

Fax: 0271 333 42 42

Email: Mohammad_golriz@yahoo.com

Index

Supplementary S1…………………………………………………………………... Page 3

Supplementary S2………………………………………………………………… Page 4

Supplementary S1- Density plot of propensity scores. Comparing the distribution of propensity scores in both groups before and after matching, which reveals a substantial overlap post-matching.

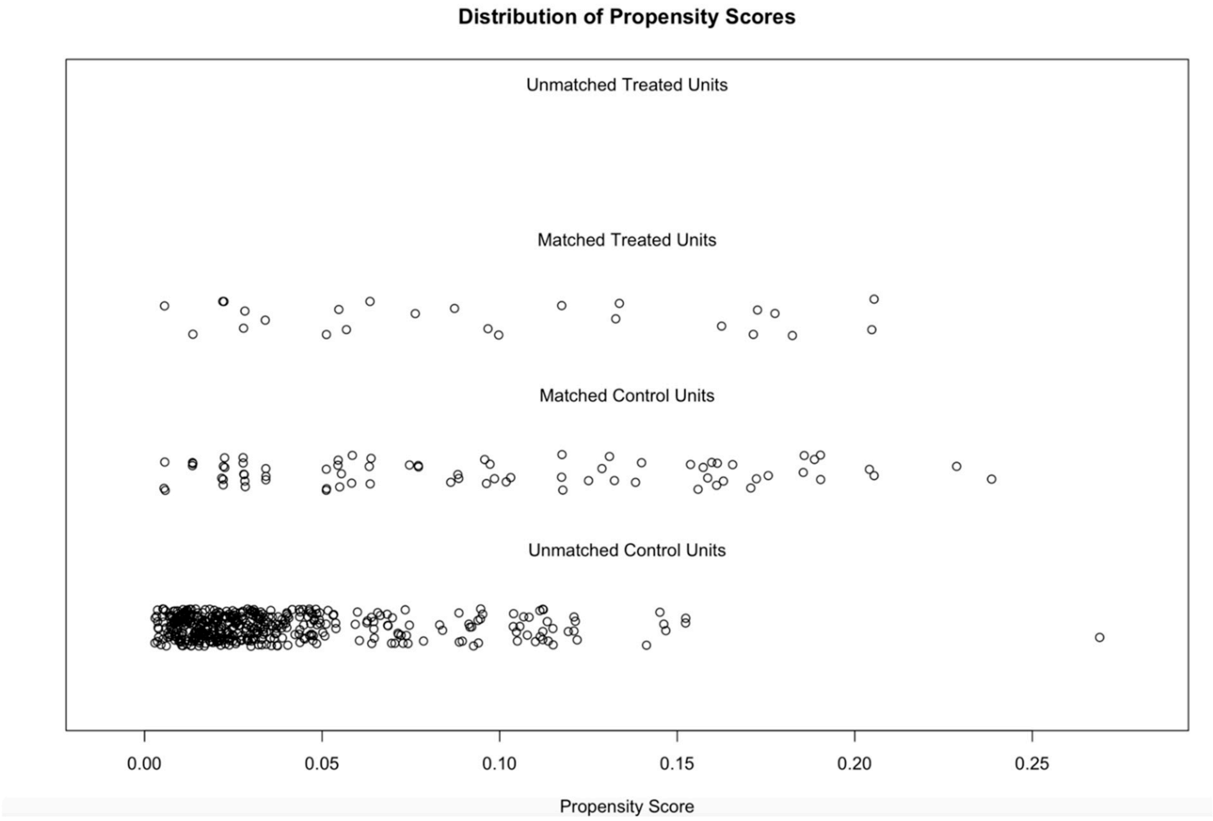

Supplementary S2- Standardized mean differences (SMD) before and after matching between groups with and without clip fenestrations. The standardized mean difference compares the means of the covariates between treated and control groups. As shown, after matching, the SMD for each covariate lies below the threshold (0.25)

	Means before matching			*Means after matching*		
	All	Treatd	Standardized Mean Differences (SMD)	Control	Treatd	Standardized Mean Differences (SMD)
Distance	0.0439	0.096	0.7964	0.953	0.096	0.0112
Age	49.8175	43.92	-0.4054	41.7467	43.92	0.1494
Gender	0.6214	0.72	0.2197	0.72	0.72	0
ASA score	2.2144	1.962	0.9402	1.9239	1.962	0.1158
Body mass index (BMI)	25.2386	24.1725	-0.2435	24.3177	24.1725	-0.0332
Previous abdominal surgery	9.2583	0.64	0.7953	0.64	0.64	0
Donors' age	55.9573	59	0.255	57.88	59	0.0939
